# Independent and synergistic effects of sedentary lifestyle and obstructive sleep apnea on hyperuricemia: a nationwide cross-sectional study in Korea

**DOI:** 10.3389/fpubh.2026.1854396

**Published:** 2026-07-15

**Authors:** Hyewon Lee, Wanhyung Lee, Seung-Yeon Lee

**Affiliations:** 1 College of Medicine, Chung-Ang University, Seoul, Republic of Korea; 2 Department of Preventive Medicine, College of Medicine, Chung-Ang University, Seoul, Republic of Korea; 3 Department of Family Medicine, International Healthcare Center, Seoul National University Bundang Hospital, Seongnam-si, Gyeonggi-do, Republic of Korea

**Keywords:** cross-sectional study, hyperuricemia, interaction effect, obstructive sleep apnea, sedentary lifestyle, STOP-bang questionnaire

## Abstract

**Introduction:**

Hyperuricemia, a precursor to gout and a risk factor for various cardiometabolic and renal disorders, is influenced by a complex interplay of lifestyle, physiological, and environmental factors. This study aimed to investigate the independent and combined effects of a sedentary lifestyle and obstructive sleep apnea (OSA) on hyperuricemia.

**Methods:**

We analyzed data from 14,808 participants aged 40–80 years in the Korea National Health and Nutrition Examination Survey (2019–2022). Sedentary lifestyle was defined based on daily sedentary time, categorized as ≤6 or >6 h/day. OSA risk was assessed using the STOP-Bang questionnaire (Snoring, Tiredness, Observed apnea, blood Pressure, Body mass index, Age, Neck circumference, Gender), classifying participants into high- and low-risk groups. Hyperuricemia was defined as serum uric acid levels ≥7.0 mg/dL in males and ≥6.0 mg/dL in females. Adjusted odds ratios (AORs) and 95% confidence intervals (CIs) were calculated using multiple logistic regression, and interaction effects were evaluated through relative excess risk due to interaction (RERI), attributable proportion (AP), and synergy index (SI).

**Results:**

Sedentary time [AOR = 1.16 (95% CI: 1.04–1.29)] was independently associated with increased hyperuricemia risk. High OSA risk was associated with hyperuricemia in the partially adjusted model [AOR = 1.94 (95% CI: 1.71–2.19)], although this association was attenuated and no longer statistically significant after further adjustment for variables incorporated into the STOP-Bang questionnaire [AOR = 1.12 (95% CI: 0.98–1.28)]. A significant synergistic interaction was evident when both coexisted [RERI = 0.14 (95% CI: 0.08–0.19), AP = 0.17 (95% CI: 0.09–0.25), SI = 1.17 (95% CI: 1.01–1.37)].

**Discussion:**

These findings highlight the need for integrated screening and management strategies targeting sedentary lifestyles and OSA to mitigate hyperuricemia and its associated health burdens in at-risk populations.

## 1 Introduction

Hyperuricemia, characterized by elevated serum uric acid levels (≥7.0 mg/dL in males and ≥6.0 mg/dL in females), is a metabolic condition that extends beyond its well-known association with gout (1). Increased uric acid accumulates in blood vessels and tissues within the body, including the heart, brain, and kidneys. A well-known example is gout, a condition in which uric acid crystals form in the joints and cause inflammation (2). Additionally, it has been increasingly implicated in a range of cardiometabolic and renal disorders, including hypertension, cardiovascular disease, and chronic kidney disease (3, 4). Despite preventive measures, the prevalence of hyperuricemia is increasing globally, driven by shifts in lifestyle, dietary patterns, and aging populations (5).

Hyperuricemia develops through an imbalance between uric acid production and excretion. The kidneys play a central role in maintaining uric acid homeostasis, with approximately two-thirds of uric acid eliminated through renal excretion (6). Consequently, impaired kidney function and altered renal urate handling are critical mechanisms underlying hyperuricemia. Increasing evidence suggests that metabolic disturbances, oxidative stress, inflammation, and endothelial dysfunction may influence both uric acid production and renal urate excretion (5–7).

A sedentary lifestyle, defined as extended periods of low physical activity, has been associated with increased risks of metabolic syndrome, obesity, cardiovascular diseases, and chronic kidney disease, all of which are linked to hyperuricemia (8, 9). Similarly, OSA, a common sleep disorder characterized by intermittent hypoxia and sleep fragmentation, has been associated with oxidative stress, systemic inflammation, and renal dysfunction, which may contribute to impaired uric acid metabolism (10, 11).

Accumulating evidence suggests that sedentary behavior and OSA may influence uric acid metabolism through multiple overlapping biological pathways, including oxidative stress, inflammation, endothelial dysfunction, metabolic dysregulation, and impaired renal urate handling (5, 10, 11). However, previous studies have largely evaluated these factors independently, and little is known about whether their coexistence confers additional risk beyond their individual effects. Given that sedentary behavior and OSA frequently coexist in middle-aged and older adults, understanding their combined contribution to hyperuricemia may provide important insights into the identification of high-risk populations and the development of integrated prevention strategies. Therefore, this study aimed to investigate the independent and interactive effects of sedentary behavior and OSA on hyperuricemia risk using nationally representative data from the Korea National Health and Nutrition Examination Survey (KNHANES).

## 2 Materials and methods

### 2.1 Data collection and study participants

The study utilized data from the eighth and ninth waves (2019–2022) of the KNHANES, a survey conducted by the Korea Disease Control and Prevention Agency (KDCA) to evaluate the health and nutritional status of the Korean population. Since 1998, KNHANES has collected data through interviews, health examinations, and nutrition assessments, providing comprehensive information on demographics, socioeconomic status, health behaviors, biochemical markers, and dietary intake. The survey employs a stratified, multistage, clustered probability sampling design to ensure national representation of individuals aged 1 year and older (12, 13).

**Figure 1:**
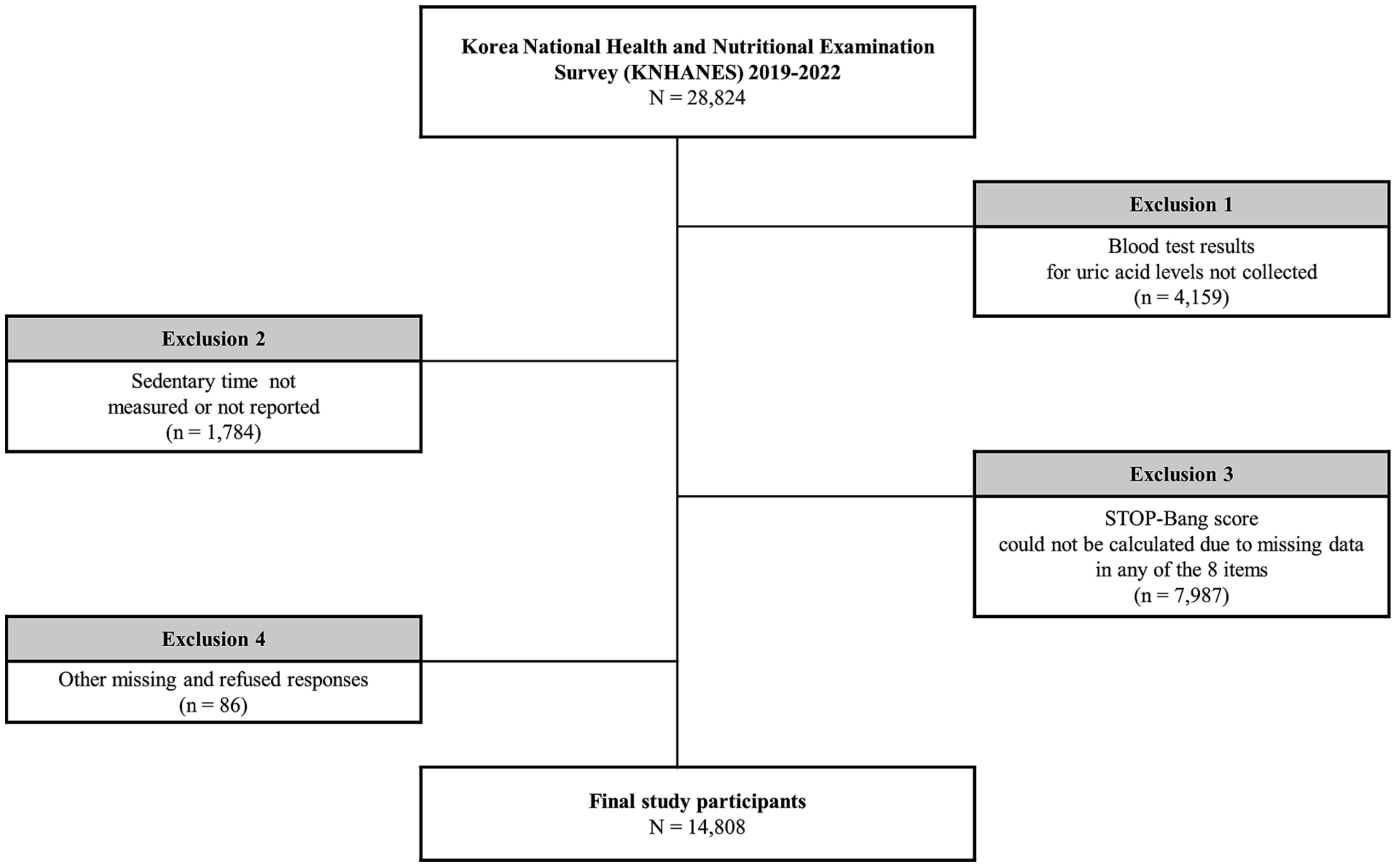
Flow chart of participant selection. Of the initial 28,824 participants aged 40 years and older in the Korea National Health and Nutrition Examination Survey (KNHANES) from 2019 to 2022, participants with missing data on serum uric acid levels (*n* = 4,159), sedentary time (*n* = 1,784), STOP-Bang questionnaire components (*n* = 7,987), and other essential variables (*n* = 86) were sequentially excluded. The final analytic sample included 14,808 participants.

Flow chart of the participant selection process is shown in Figure 1. Among the 28,824 participants with data from 2019 to 2022, only those available for assessment of sedentary time and OSA were included. The STOP-Bang questionnaire was newly introduced in the 8th wave of KNHANES (2019) and was administered only to participants aged 40 years and older. Therefore, individuals aged under 40 years were excluded from the analysis. Participants with missing data on serum uric acid levels, socioeconomic variables (age, sex, education level, and household income), or health-related variables (smoking status, alcohol consumption, body mass index (BMI), aerobic exercise, hypertension, diabetes mellitus, and renal disease) were also excluded. No imputation was performed for missing data; only participants with complete data on all relevant variables were included in the final analysis. A total of 14,808 participants were included in the final analysis.

This study was conducted in accordance with the principles of the Declaration of Helsinki. The study protocol was reviewed and approved by the Institutional Review Board of the Korea Disease Control and Prevention Agency (IRB approval numbers: 2018-01-03-C-A, 2018-01-03-2C-A, 2018-01-03-5C-A, and 2018-01-03-4C-A). The analysis was based on publicly available KNHANES data, which had been anonymized and de-identified prior to release. All participants provided written informed consent during the original data collection. As a secondary data analysis of de-identified data, this study did not require additional ethical approval or consent.

### 2.2 Sedentary lifestyle

To investigate sedentary lifestyles, we used data from self-report questionnaires on daily sedentary time. Sedentary time was defined based on responses to questions regarding time spent sitting or lying down while working, at home, moving around, or socializing with friends, excluding sleep duration. Those with a sedentary time of 6 h or less were classified as relatively active during the daytime, whereas those with a sedentary time exceeding 6 h were classified as having a relatively sedentary lifestyle, following the approach of Oh et al. who applied the same KNHANES-based sedentary time variable in a Korean adult population (14). We acknowledge that the optimal cut-point for sedentary time in relation to hyperuricemia has not been firmly established in the literature, and future studies employing dose–response analyses would help clarify the most appropriate threshold.

### 2.3 OSA

The risk for OSA was assessed using the STOP-Bang questionnaire, a validated screening tool comprising four self-reported items (snoring, tiredness, observed apnea, high blood pressure) and four demographic items (BMI, age, neck circumference, sex) (15, 16). Although polysomnography (PSG) is the diagnostic gold standard, STOP-Bang is more feasible for large-scale epidemiological studies due to its lower cost and convenience (17, 18). One point was assigned for each of the following conditions: snoring, daytime tiredness, stopped breathing while sleeping, high blood pressure, BMI > 35 kg/m^2^, age > 50 years, neck circumference > 40 cm, and male sex. Individuals scoring ≥5 were classified as high risk. Those scoring 3–4 were also considered high risk if they met ≥2 STOP items and had either BMI > 35 kg/m^2^, neck circumference >40 cm, or were male. Others were classified as low risk. In KNHANES, STOP-Bang was administered only to participants aged 40 years and older (19–21). In the 8th wave of the KNHANES, the STOP-Bang score was first surveyed only for people aged 40 years and older.

### 2.4 Hyperuricemia

Hyperuricemia was defined as serum uric acid levels of 7.0 mg/dL or higher in males and 6.0 mg/dL or higher in females (1, 22, 23). Laboratory tests were performed to measure the uric acid levels (mg/dL). Venous blood samples were drawn from the participants by trained medical personnel and analyzed in a certified laboratory operating under external quality control programs. Serum uric acid levels were measured by colorimetric determination with a uricase–catalase system (Hitachi Automatic Analyzer 7,600–210; Hitachi, Tokyo, Japan) (24).

### 2.5 Covariates

The socioeconomic variables were sex, age, educational level, and household income. Age was classified into four groups: 40s, 50s, 60s, and 70s, with 80-year-olds included in the final group. Educational level was classified into four groups: elementary school graduate or lower, middle school graduate, high school graduate, and college graduate or higher. Household income was classified into quartiles: low, middle-low, middle-high, and high. The health-related variables were smoking status, alcohol consumption, BMI, and aerobic exercise. Smoking status was dichotomized into current smokers and non-smokers, the latter encompassing former smokers and those who had never smoked. Alcohol consumption was categorized based on the Alcohol Use Disorders Identification Test-Korean Revised Version (AUDIT-K). Heavy drinkers were defined as those who consumed, on average, seven or more drinks per occasion for men and five or more drinks per occasion for women, with a frequency of at least twice weekly. Those who did not meet these criteria were classified as non-heavy drinkers (25). BMI was classified using Asian criteria: < 18.5 kg/m^2^, between 18.5 kg/m^2^ and 25 kg/m^2^, and > 25 kg/m^2^ (26). Aerobic exercise, such as running, cycling, or swimming, is recommended for managing hyperuricemia. Therefore, aerobic activity was included as a covariate and classified as sufficient or insufficient. The sufficient group consisted of individuals who engaged in at least 2 h and 30 min of moderate-intensity activity, at least 1 h and 15 min of vigorous-intensity activity per week, or an equivalent combination (1 min of vigorous activity considered equal to 2 min of moderate activity). All others were classified as insufficient (27). Hypertension, diabetes mellitus, and renal disease were additionally included as covariates, as these conditions frequently co-occur with hyperuricemia within the broader context of cardiometabolic dysregulation. In particular, hypertension and renal disease have been independently associated with impaired renal urate excretion and elevated serum uric acid levels, and diabetes mellitus clusters closely with other components of metabolic syndrome (28). Each variable was assessed based on self-reported physician diagnosis and categorized as present or absent.

### 2.6 Statistical analysis

To examine the overall association between each variable and hyperuricemia, a chi-square test was conducted. We obtained adjusted odds ratios (AORs) and 95% confidence intervals (CIs) for sedentary time and OSA for the risk of hyperuricemia using multiple logistic regression analyses. Crude model was unadjusted. Model 1 was adjusted for education level, household income, smoking status, alcohol consumption, aerobic exercise, diabetes, and renal disease. Model 2 was additionally adjusted for age, sex, body mass index, and hypertension, which are variables incorporated into the STOP-Bang questionnaire. This hierarchical approach was adopted to demonstrate how the association changes with progressive adjustment for potential confounders, including variables incorporated into the STOP-Bang questionnaire. To explore potential non-linear relationships between serum uric acid levels and the study exposures, we modelled daily sedentary time and STOP-Bang score as continuous dependent variables using generalized additive models. Daily sedentary time (hours) and STOP-Bang score were each regressed on serum uric acid level (mg/dL) with a penalized spline term for uric acid. From these models, we obtained predicted values and 95% CIs across the observed range of serum uric acid. The interactive effects of sedentary time and OSA on hyperuricemia were estimated using the relative excess risk due to interaction (RERI), attributable proportion due to interaction (AP), and synergy index (SI), based on age-standardized prevalence ratios (SPR). The RERI has been widely used to assess the additivity of effects on the relative risk (RR). Although a well-known RERI formula exists, this interactive analysis used an indirect standardization method based on an age-adjusted standardized prevalence ratio. Therefore, a modified formula was applied to calculate RERI. The AP was interpreted as the proportion of disease due to the interaction between both exposures. The SI was interpreted as the excess risk from exposure to both exposures when there is an interaction, relative to the risk from exposure without interaction. The formula for the SI was modified for the same reasons as for the RERI. If there is an absence of interaction, RERI and AP are both equal to zero, and SI equals one. When the values of RERI or AP were greater than 0, or AP exceeded 1, this indicated a positive interaction effect, which was considered significant if the 95% CIs did not contain 0. $RR_{++}$ denotes RR when both risk factors were present. $RR_{+-}$ denotes the RR when only one risk factor is present and the other is absent. The same principle applies to denote $RR_{-+}$ and $RR_{--}$ . The equation for RERI, AP, and SI are as follows:


$$\text{RERI}=RR_{++}-RR_{+-}-RR_{-+}+RR_{--}$$

$$\mathrm{AP}=\text{RERI}/RR_{++}$$

$$\mathrm{SI}=[RR_{++}-RR_{--}]/[(RR_{+-}-RR_{--})+(RR_{-+}-RR_{--})]$$

All statistical analyses were performed using SAS version 9.4. software (SAS Institute, Cary, NC, USA). All *p*-values were two-tailed, and values of *p* < 0.05 were considered statistically significant. All analyses incorporated sampling weights and accounted for the complex survey design of KNHANES to ensure nationally representative estimates.

## 3 Results

Table 1 shows the descriptive characteristics of participants with hyperuricemia. Among the 14,808 participants, 1,824 (12.3%) had hyperuricemia. Significant differences were observed between participants with and without hyperuricemia across various characteristics, including sex, age, smoking status, alcohol consumption, BMI, hypertension, and renal disease. Additionally, hyperuricemia was more prevalent among individuals with sedentary time exceeding 6 h per day and those identified as being at high risk for OSA.

**Table 1:** Descriptive characteristics of participants with hyperuricemia.

Characteristics	Total	Hyperuricemia		*p*-value
		No (%)	Yes (%)	
Overall	14,808 (100.0)	12,984 (87.7)	1,824 (12.3)	
Sex				< 0.0001
Men	6,460 (43.6)	5,302 (82.1)	1,158 (17.9)	
Women	8,348 (56.4)	7,682 (92.0)	666 (8.0)	
Age (years)				< 0.0001
40–49	3,548 (23.9)	3,036 (85.6)	512 (14.4)	
50–59	3,793 (25.6)	3,389 (89.3)	404 (10.7)	
60–69	4,007 (27.1)	3,584 (89.4)	423 (10.6)	
70–80	3,460 (23.4)	2,975 (86.0)	485 (14.0)	
Education level				0.6926
Elementary school	3,392 (22.9)	2,956 (87.2)	436 (12.8)	
Middle school	1,821 (12.3)	1,589 (87.3)	232 (12.7)	
High school	4,822 (32.6)	4,280 (88.8)	542 (11.2)	
College or higher	4,773 (32.2)	4,159 (87.1)	614 (12.9)	
Household income				0.0608
Low	3,165 (21.4)	2,724 (86.1)	441 (13.9)	
Middle-low	3,725 (25.2)	3,287 (88.2)	438 (11.8)	
Middle-high	3,798 (25.6)	3,359 (88.4)	439 (11.6)	
High	4,120 (27.8)	3,614 (87.7)	506 (12.3)	
Smoking status				< 0.0001
Non-smokers	12,573 (84.9)	11,147 (88.7)	1,426 (11.3)	
Current smokers	2,235 (15.1)	1,837 (82.2)	398 (17.8)	
Alcohol consumption				< 0.0001
Non-heavy drinkers	13,222 (89.3)	11,764 (89.0)	1,458 (11.0)	
Heavy drinkers	1,586 (10.7)	1,220 (76.9)	366 (23.1)	
Body mass index (kg/m^2^)				< 0.0001
< 18.5	380 (2.6)	359 (94.5)	21 (5.5)	
18.5–25	8,879 (59.9)	8,057 (90.7)	822 (9.3)	
≥ 25	5,549 (37.5)	4,568 (82.3)	981 (17.7)	
Aerobic exercise				0.4666
Insufficient	8,969 (60.6)	7,850 (87.5)	1,119 (12.5)	
Sufficient	5,839 (39.4)	5,134 (87.9)	705 (12.1)	
Hypertension				< 0.0001
No	9,789 (66.1)	8,763 (89.5)	1,026 (10.5)	
Yes	5,019 (33.9)	4,221 (84.1)	798 (15.9)	
Diabetes				0.9326
No	12,723 (85.9)	11,157 (87.7)	1,566 (12.3)	
Yes	2,085 (14.1)	1,827 (87.6)	258 (12.4)	
Renal disease				< 0.0001
No	14,580 (98.5)	12,807 (87.8)	1,773 (12.2)	
Yes	228 (1.5)	177 (77.6)	51 (22.4)	
Sedentary time (daily)				0.0026
≤ 6 h	5,109 (34.5)	4,537 (88.8)	572 (12.2)	
> 6 h	9,699 (65.5)	8,447 (87.1)	1,252 (12.9)	
Obstructive sleep apnea				< 0.0001
Low risk	12,684 (85.7)	11,315 (89.2)	1,369 (10.8)	
High risk	2,124 (14.3)	1,669 (78.6)	455 (21.4)	

Table 2 presents the associations of sedentary time and OSA risk with hyperuricemia. Participants with sedentary time exceeding 6 h per day had a significantly higher odds of hyperuricemia compared to those with sedentary time of 6 h or less (Model 2: AOR = 1.16, 95% CI: 1.04–1.29, *p* = 0.010). Participants at high risk for OSA had increased odds of hyperuricemia in the partially adjusted model (Model 1: AOR = 1.94, 95% CI: 1.71–2.19, *p* < 0.001), although this association was attenuated and no longer statistically significant after full adjustment (Model 2: AOR = 1.12, 95% CI: 0.98–1.28, *p* = 0.103). These findings suggest that prolonged sedentary time was independently associated with hyperuricemia after adjustment for multiple confounding factors, whereas the association between OSA risk and hyperuricemia was largely explained after further adjustment for variables incorporated into the STOP-Bang questionnaire.

**Table 2:** Association of sedentary time and the risk for obstructive sleep apnea with hyperuricemia.

	Odd ratio (95% confidence interval) for hyperuricemia, *p*-value		
	Crude	Model 1	Model 2
Sedentary time (daily)			
≤ 6 h	1.00 (reference)	1.00 (reference)	1.00 (reference)
> 6 h	1.18 (1.06–1.31), 0.003	1.20 (1.08–1.33), <0.001	1.16 (1.04–1.29), 0.010
Obstructive sleep apnea			
Low risk	1.00 (reference)	1.00 (reference)	1.00 (reference)
High risk	2.25 (2.00–2.54), <0.001	1.94 (1.71–2.19), <0.001	1.12 (0.98–1.28), 0.103

Crude: unadjusted model.

Model 1: adjusted for education level, household income, smoking status, alcohol consumption, aerobic exercise, diabetes, and renal disease.

Model 2: Model 1 additionally adjusted for age, sex, body mass index, and hypertension.

OR, odds ratio; CI, confidence interval; OSA, obstructive sleep apnea.

**Figure 2:**
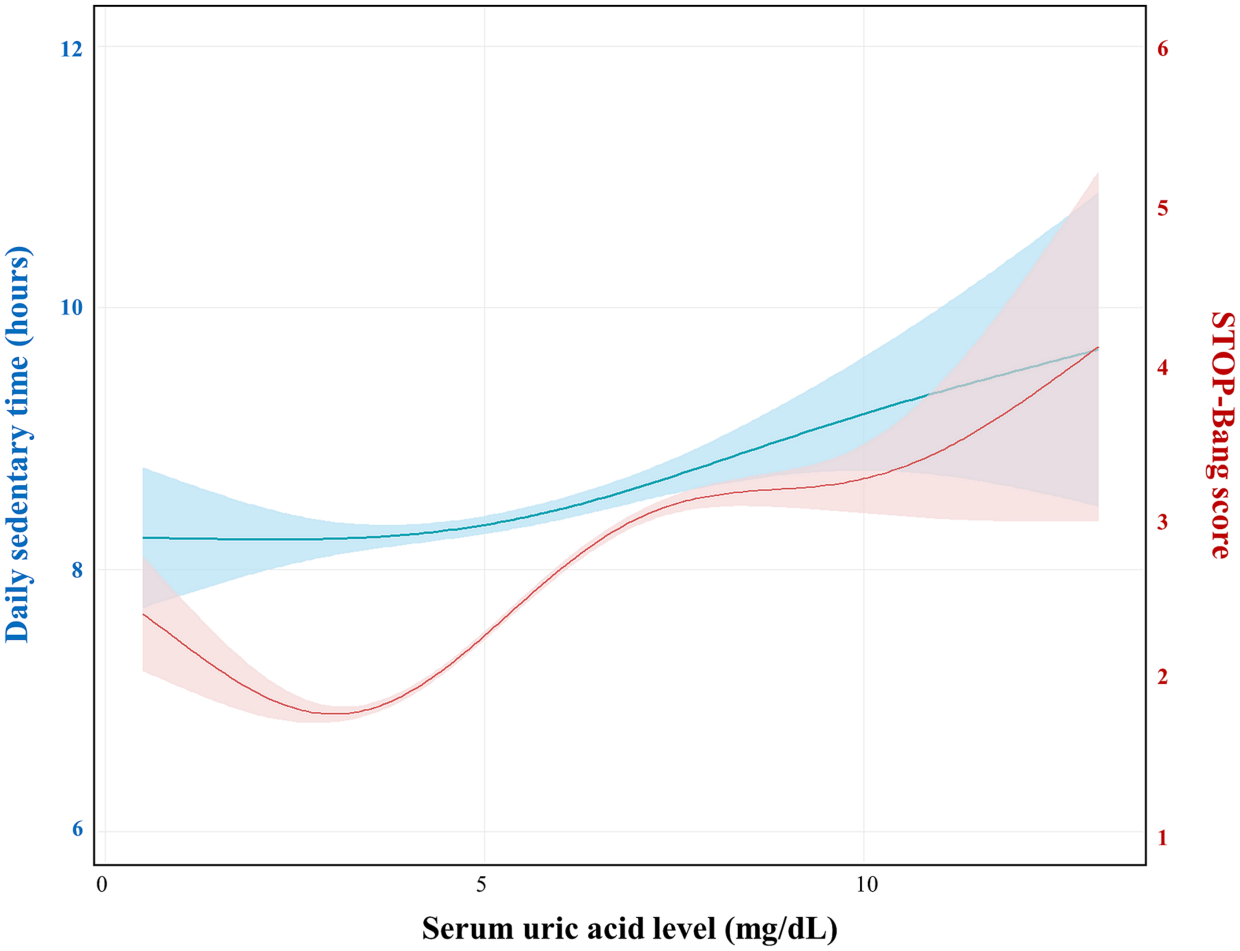
Trends of sedentary time and STOP-Bang score according to serum uric acid levels. Spline-based predicted trends of daily sedentary time (left *y*-axis, blue line and shaded area) and STOP-Bang score (right *y*-axis, red line and shaded area) across the distribution of serum uric acid levels. Lines represent predicted means from generalized additive models and shaded bands indicate 95% confidence intervals.

Figure 2 depicts the non-linear trends of daily sedentary time and STOP-Bang score across the distribution of serum uric acid levels. Overall, both sedentary time and STOP-Bang score increased with higher serum uric acid. At lower uric acid levels, the predicted sedentary time was relatively flat around 8 h/day and then rose gradually as uric acid exceeded approximately 6–7 mg/dL, reaching more than 10 h/day at the upper end of the uric acid distribution. In contrast, the STOP-Bang score showed a modest dip at intermediate uric acid levels and then increased steeply at higher uric acid levels, with scores exceeding 4 points at uric acid levels above about 9–10 mg/dL. The 95% confidence bands widened toward the extremes of the uric acid distribution, reflecting the smaller number of participants in these tails.

**Figure 3:**
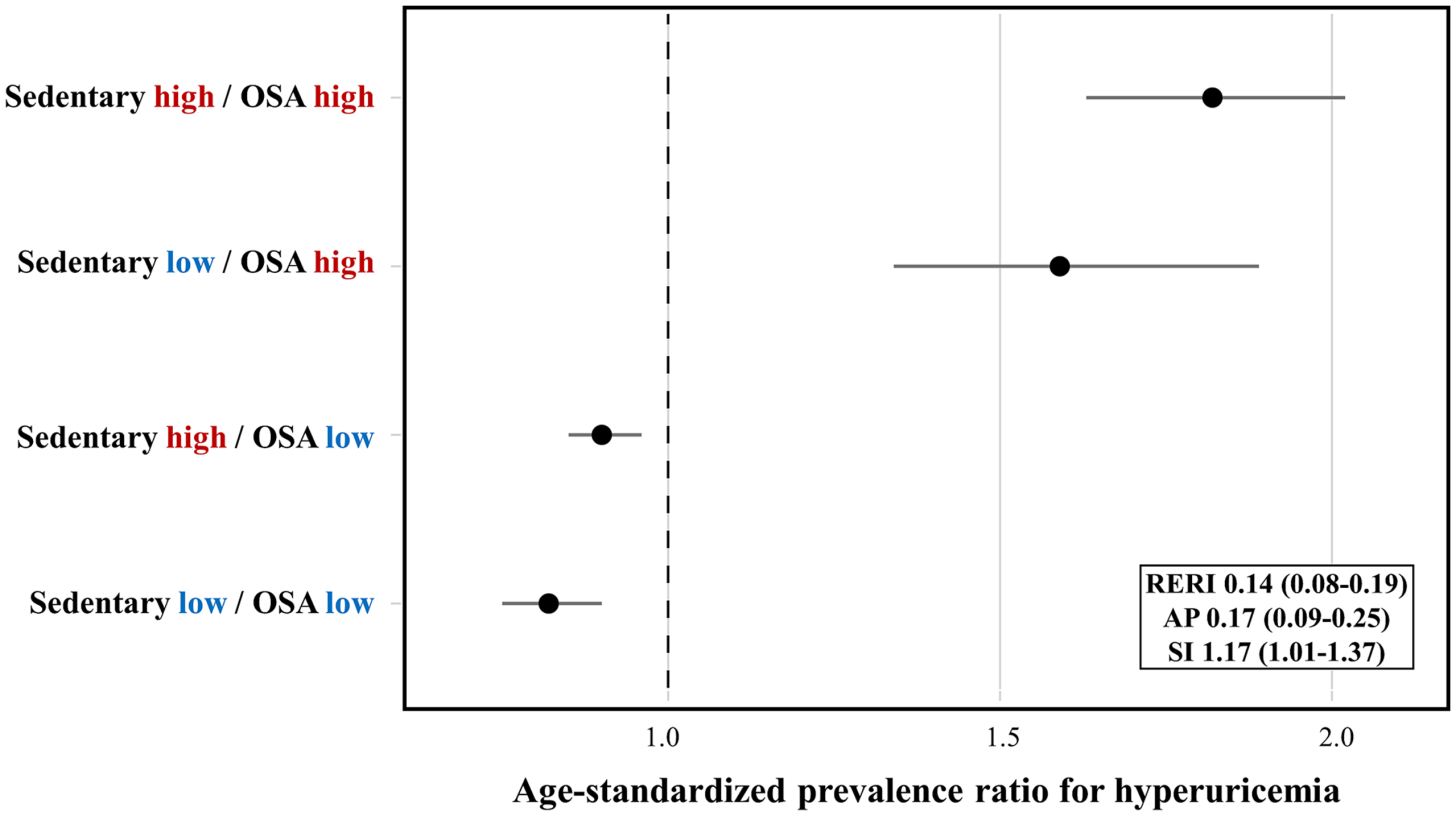
Age-standardized prevalence ratios between sedentary time and obstructive sleep apnea risk for hyperuricemia. Forest plot of age-standardized prevalence ratios (SPRs) for hyperuricemia according to combinations of sedentary time (low vs. high) and obstructive sleep apnea (OSA) risk (low vs. high). The reference group is low sedentary time and low OSA risk (SPR = 1.00). Dots indicate point estimates and horizontal lines 95% confidence intervals. The box displays additive interaction indices for the joint exposure to high sedentary time and high OSA risk versus the other combinations: relative excess risk due to interaction (RERI), attributable proportion due to interaction (AP), and synergy index (SI).

Figure 3 illustrates the interaction between sedentary time and OSA risk on hyperuricemia. The relative excess risk due to interaction (RERI) was 0.14 (95% CI: 0.08–0.19), the attributable proportion (AP) due to interaction was 0.17 (95% CI: 0.09–0.25), and the synergy index (SI) was 1.17 (95% CI: 1.01–1.37), indicating a potential synergistic effect of prolonged sedentary time and high OSA risk on hyperuricemia.

The fitted 3D trend surface of serum uric acid according to STOP-Bang score and sedentary time (Supplementary Figure S1) visually confirmed that the overall associations were approximately linear across the observed ranges.

## 4 Discussion

This study demonstrated that a sedentary lifestyle and OSA are independently associated with an increased risk of hyperuricemia. Furthermore, their coexistence was associated with a statistically significant additive interaction, although the magnitude of interaction was modest. These findings highlight the importance of considering the combined impact of behavioral and physiological factors on metabolic disorders such as hyperuricemia.

Several biological mechanisms may explain the observed interaction between sedentary behavior and OSA. Hyperuricemia results from an imbalance between uric acid production and excretion, and both exposures may influence these pathways simultaneously (5, 6). Because kidneys are responsible for the majority of uric acid elimination, impaired renal function and altered renal urate handling are considered key mechanisms underlying hyperuricemia (6, 7). In addition, growing evidence suggests that oxidative stress, inflammation, endothelial dysfunction, and metabolic disturbances may influence both uric acid production and renal urate excretion (5, 7, 29, 30).

OSA may contribute to hyperuricemia through several interconnected pathways. Recurrent intermittent hypoxia accelerates adenosine triphosphate degradation and purine catabolism, resulting in increased uric acid production (31). Intermittent hypoxia has also been shown to activate xanthine oxidase and promote oxidative stress and systemic inflammation, further contributing to urate overproduction and renal injury (29, 32, 33). Moreover, OSA-related sympathetic activation, endothelial dysfunction, and hemodynamic alterations may impair renal blood flow and glomerular filtration, thereby reducing uric acid clearance (11, 34–36).

Sedentary behavior may further amplify these effects through metabolic and vascular dysregulation. Prolonged sedentary time is associated with insulin resistance, obesity, and chronic low-grade inflammation (37). Insulin resistance may increase renal tubular urate reabsorption through altered activity of urate transporters, including URAT1 and GLUT9, leading to elevated serum uric acid concentrations (38). Sedentary behavior has also been associated with endothelial dysfunction and impaired vascular responsiveness, which may adversely affect renal perfusion and kidney function (8). Furthermore, prolonged physical inactivity may contribute to chronic kidney disease development and progression, thereby reducing renal urate excretion capacity (30, 37).

Importantly, these mechanisms are unlikely to operate independently. OSA may primarily increase uric acid production through intermittent hypoxia, enhanced purine metabolism, and oxidative stress, whereas sedentary behavior may predominantly promote urate retention through insulin resistance, vascular dysfunction, and impaired renal urate handling. Both conditions are also associated with systemic inflammation, endothelial dysfunction, and renal impairment. Therefore, the coexistence of sedentary behavior and OSA may be associated with alterations in both uric acid production and excretion. Although these pathways provide a biologically plausible explanation for the observed additive interaction, the present findings should be interpreted as associations rather than evidence of a direct causal mechanism. The observed interaction may reflect the influence of shared metabolic and renal physiological pathways. However, given the modest magnitude of the interaction and the cross-sectional design, these proposed mechanisms remain speculative and require confirmation in prospective and mechanistic studies.

We also identified several significant covariates influencing hyperuricemia, including sex, age, smoking, alcohol consumption, and BMI. These findings align with established literature, notably Krishnan et al.’s longitudinal analysis of the Framingham Offspring Study, which demonstrated these factors as significant hyperuricemia predictors (39). However, socioeconomic factors such as income and education were not significantly associated with hyperuricemia in our study, consistent with Pan et al.’s systematic review and meta-analysis in high-income countries including the United States and South Korea (40). Historically, gout, caused by hyperuricemia, was often referred to as the “disease of kings,” reflecting the belief that high uric acid levels were linked to wealth and higher socioeconomic status. However, advances in food processing and the widespread availability of purine-rich fast food have made such diets accessible across all income levels. Additionally, individuals with higher education often adopt healthier lifestyles and diets, reducing their exposure to hyperuricemia risk factors. Moreover, greater healthcare access and more frequent screenings in higher socioeconomic groups may also increase detection of asymptomatic hyperuricemia. Given these factors, the lack of a significant association between socioeconomic status and hyperuricemia in our study aligns with contemporary findings and may reflect broader changes in dietary habits and healthcare access. Similarly, our analysis found no significant association between aerobic exercise and hyperuricemia. This aligns with Choi et al.’s study on modifiable risk factors using NHANES-III data, which identified BMI as the strongest predictor. After adjusting for BMI, the association between exercise and hyperuricemia disappeared, suggesting that exercise may influence uric acid levels indirectly through its effect on BMI rather than through a direct mechanism (41).

First, the analysis was restricted to Korean adults aged 40 years and older. Therefore, the findings may not be generalizable to younger age groups or to populations from different ethnic or regional backgrounds. In addition, because the final analytic sample was derived through complete-case analysis, some degree of selection bias cannot be excluded, and the findings should be interpreted with caution when extrapolating to populations with substantially different demographic or metabolic characteristics. However, hyperuricemia and its clinical manifestations, such as gout, are most prevalent among those aged 45 and older (39), making this age group particularly relevant. Second, although the STOP-Bang questionnaire is a practical and widely validated screening tool for OSA risk in large-scale epidemiological studies, it should not be interpreted as a clinical diagnosis of OSA (17). Its applicability to middle-aged and older Korean adults also warrants caution. Because STOP-Bang assigns points for male sex and age over 50 years, older men may be more likely to receive higher scores irrespective of symptom burden, potentially contributing to overclassification of OSA risk in this subgroup (42). Furthermore, as the STOP-Bang questionnaire was originally developed and validated in Western populations (15), the original BMI and neck circumference thresholds may not be optimally calibrated for Korean adults; Korean validation studies have suggested that lower anthropometric cutoffs may be more appropriate for screening South Korean patients with OSA (43, 44). Moreover, the performance of STOP-Bang has been reported to vary across geographic regions, with lower diagnostic accuracy observed in East Asian cohorts (17). Therefore, STOP-Bang–based OSA risk classification in this study should be interpreted as a screening-based estimate of OSA risk rather than a confirmed diagnosis. Nonetheless, STOP-Bang remains a practical and resource-efficient tool for population-level OSA risk stratification in large-scale studies such as KNHANES, where objective diagnostic testing with polysomnography is not feasible because of its cost, time requirements, and limited scalability. In addition, STOP-Bang has demonstrated high sensitivity and negative predictive value for identifying moderate-to-severe OSA and continues to undergo refinement to improve its diagnostic performance (17, 45, 46). Third, the cross-sectional design limits causal inference and raises the possibility of reverse causality. Individuals with hyperuricemia or gout-related symptoms may reduce physical activity because of pain, discomfort, or associated comorbidities, thereby increasing sedentary behavior. Likewise, cardiometabolic conditions commonly associated with hyperuricemia may contribute to sleep disturbances and increase the likelihood of OSA-related symptoms. Therefore, the temporal relationships among sedentary behavior, OSA, and hyperuricemia cannot be definitively established in the present study. Nevertheless, current biological and epidemiological evidence generally supports sedentary behavior and OSA as potential risk factors for hyperuricemia rather than the reverse relationship. Prospective longitudinal studies are needed to clarify the direction and causality of these associations. Additionally, we could not account for some important determinants of serum uric acid, such as detailed dietary purine and fructose intake, urate lowering therapy, and specific diuretic use, because these variables were not available (or not available with sufficient detail) in the KNHANES data. Therefore, residual confounding by these unmeasured factors cannot be completely ruled out. These unmeasured variables could overestimate or mask the true associations observed, given their prevalence in middle-aged and older adult populations. Future studies should incorporate these factors to refine the estimates of sedentary lifestyle and OSA’s contributions to hyperuricemia risk.

Despite these limitations, this study has notable strengths. It utilized nationally representative data from KNHANES, enhancing generalizability to middle-aged and older Korean adults. The large sample size of 14,808 participants further increased statistical power. By examining both the independent and interactive effects of sedentary lifestyle and OSA on hyperuricemia, the study offers novel insights into their synergistic impact and addresses a key gap in existing literature. The use of validated tools, such as the STOP-Bang questionnaire and standardized hyperuricemia criteria, along with adjustment for key covariates, strengthens the robustness of the findings. By focusing on modifiable risk factors, the study has practical implications for integrated prevention strategies in both clinical and public health contexts.

## 5 Conclusion

This study, based on a representative sample of 14,808 Korean adults aged 40–80 years, demonstrated that both sedentary lifestyle and OSA were independently associated with hyperuricemia. In addition, a significant additive interaction was observed between the two exposures, suggesting that their coexistence may confer a greater risk of hyperuricemia than either factor alone; however, the magnitude of the interaction was modest. Although the underlying biological mechanisms cannot be confirmed in this cross-sectional study, several shared metabolic and renal physiological pathways may plausibly contribute to the observed association. These findings suggest that individuals with both prolonged sedentary behavior and a high risk of OSA may represent a population at particularly increased risk of hyperuricemia. From a public health perspective, reducing sedentary behavior and improving identification and management of OSA may help mitigate the burden of hyperuricemia and its related cardiometabolic complications. Future longitudinal and mechanistic studies are warranted to further clarify the temporal relationships and biological pathways underlying these associations.

## Data Availability

The data underlying this study are publicly available from KNHANES, conducted by KDCA. The datasets can be accessed through the official KNHANES website (https://knhanes.kdca.go.kr/knhanes/main.do) upon free registration and data use agreement.

## Supplementary material

SUPPLEMENTARY FIGURE S1 Three-dimensional trend surface of serum uric acid levels according to STOP-Bang score and sedentary time. The smooth, monotonically increasing surface, without prominent inflection points or plateaus, visually supports approximately linear associations between both exposures and serum uric acid levels across the observed ranges.
