# Supplementary figures and images for: Independent and synergistic effects of sedentary lifestyle and obstructive sleep apnea on hyperuricemia: a nationwide cross-sectional study in Korea
Source: Front Public Health. 2026 Jul 15;14:1854396. doi: 10.3389/fpubh.2026.1854396 (PMC13414846; doi:10.3389/fpubh.2026.1854396)

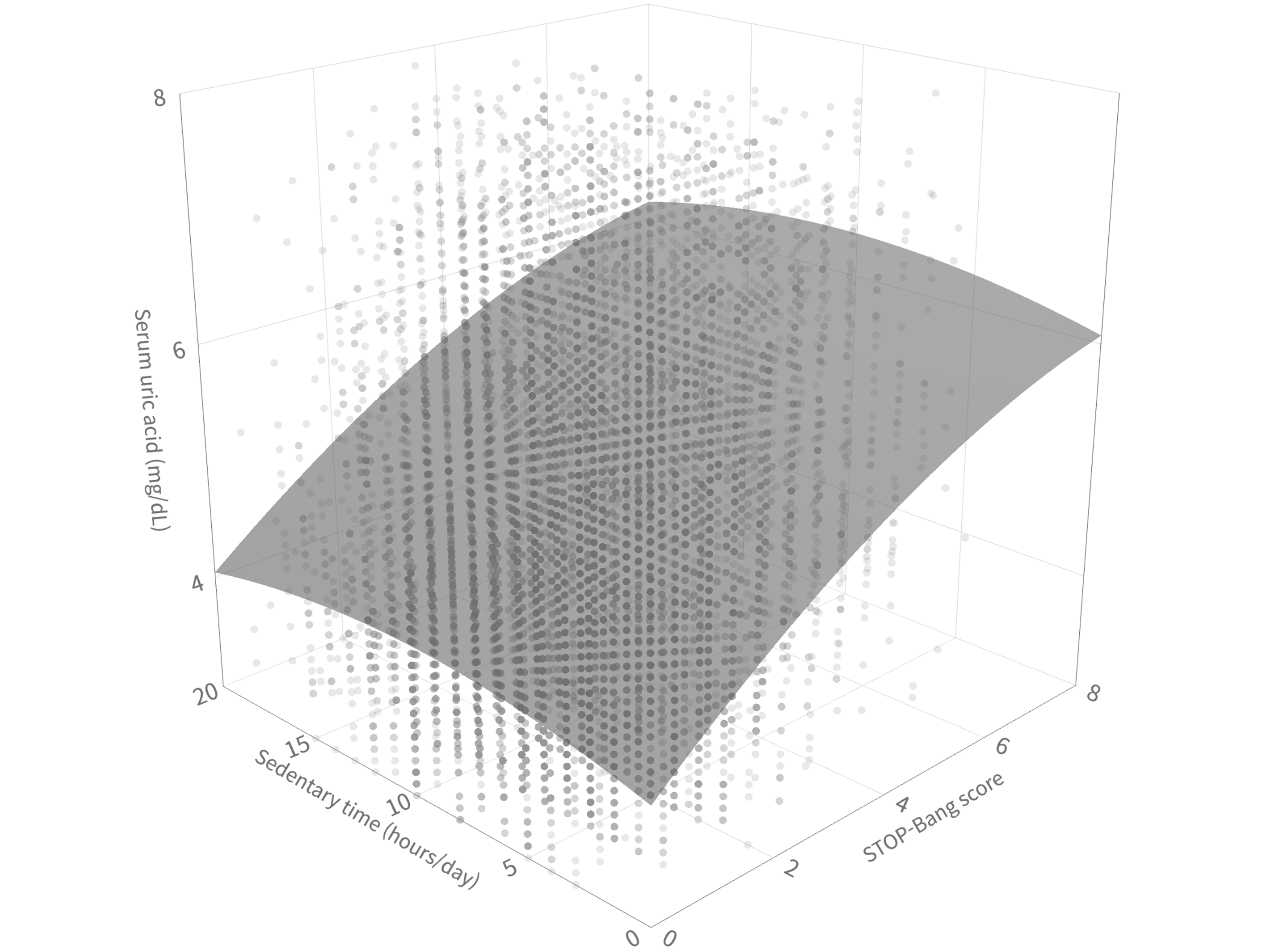

Supplement: SUPPLEMENTARY FIGURE S1 — Three-dimensional trend surface of serum uric acid levels according to STOP-Bang score and sedentary time. The smooth, monotonically increasing surface, without prominent inflection points or plateaus, visually supports approximately linear associations between both exposures and serum uric acid levels across the observed ranges. [file Image_1.TIF]
